# Water Quality Criteria of Dieldrin for the Protection of Aquatic Organisms and Wildlife Using a Tissue Residue Approach

**DOI:** 10.3390/toxics13030173

**Published:** 2025-02-27

**Authors:** Li Xie, Xuemei Li, Liangwen Bao, Yuexin Zhang, Hailei Su, Xuesong Liu, Fanfan Wang, Yuan Wei, Ningning Ji, Min Zhou

**Affiliations:** 1 State Key Laboratory of Environmental Criteria and Risk Assessment, Chinese Research Academy of Environmental Sciences, Beijing 100012, China; 2 College of Environmental Science and Engineering, Liaoning Technical University, Fuxin 123032, China

**Keywords:** dieldrin, water quality criteria, bioaccumulation, ecological risk, wildlife criteria

## Abstract

Dieldrin is legacy organochlorine insecticide, which was listed in the Stockholm Convention because of its persistence, bioaccumulation and toxicity. However, it is still present in the environment and in organisms two decades after its ban. The current criteria used for risk assessment in China are based on acute toxicity data in water columns without considering the bioavailability and bioaccumulation, which accordingly lead to the under-protection of aquatic organisms and wildlife. In this study, the water quality criteria (WQC) for dieldrin were derived from a combination of tissue-based toxicity data and the bioaccumulation factor (BAF) to better protect aquatic ecosystems. The dieldrin residue data in surface water in China were obtained by literature review and the ecological risk was assessed using the quotient method. Combined with a BAF of 58,884.37 L/kg estimated by the model, the WQC were calculated as needing to be 3.86 and 1.4 ng/L to protect aquatic life and aquatic-dependent wildlife, respectively. The results of the risk assessment revealed the potential high risk posed by dieldrin bioaccumulation. This study provides scientific guidance for the determination of the water quality standard for dieldrin and to ensure the risk management of the aquatic environment in China.

## 1. Introduction

Dieldrin (C12H8Cl6O) is both a synthetic organochlorine pesticide (OCP) classified as a chlorinated cyclodiene compound that was primarily used on cotton, corn, and citrus crops and an environmental and metabolic product of the related compound aldrin [1]. Dieldrin has a low water solubility of 0.110 mg/L at 20 °C [2]. It is lipophilic and persistent in soil has a half-life of up to five years. Dieldrin possesses high potential for bioaccumulation and readily bioaccumulates in terrestrial and aquatic organisms [3,4,5,6], tending to bio-magnify in the food chain.

The endpoints of dieldrin have been identified, including its carcinogenicity, endocrine disruptions, reproductive/development effects and neurotoxicity [7,8,9,10]. Due to its adverse effects on humans and the ecosystem, dieldrin’s persistence in the environment has become a major concern, prompting its inclusion as one of the 12 initial persistent organic pollutants (POPs) under the Stockholm Convention, which imposed a ban on its manufacturing, usage and commerce worldwide. However, dieldrin has been detected worldwide in the environment through studies related to pesticides [11,12,13,14,15]. Its residue has also been monitored in human milk [16,17,18] and wildlife, such as in raptors across the world [19] and in whales and polar bears in the Arctic [20].

Dieldrin is very toxic to aquatic life with long lasting effects and is suspected of causing cancer. Some guidelines and standards have been set for dieldrin. In Canada, water quality guideline of the long-term concentration for the protection of aquatic life was first published in 1987 but is no longer recommended because environmental exposure predominantly occurs via sediment, soil and/or tissue. Only sediment quality guidelines for the protection of aquatic life are recommended. CCME developed interim sediment quality guidelines for Canada regarding the probable effect levels for dieldrin, which can be used to assess the likelihood that exposure to dieldrin in sediments will have detrimental biological effects [21]. The USEPA released the procedures to obtain dieldrin equilibrium partitioning sediment benchmarks, in order to protect benthic organisms [22]. The USEPA does not provide the ambient water quality criterion for dieldrin. But the wildlife value for dieldrin in waters within the Great Lakes Basin was calculated as 0.071 ng/L, based on field measurements and trophic level-specific factors [23]. In the European Union, the environmental quality standard (EQS) value for dieldrin, namely 10 ng/L, is used to evaluate the pollution of rivers [24]. Moreover, the EQS value has been amended to 10 ng/L for cyclodiene pesticides (including aldrin, dieldrin endrin, and isodrin) because these substances are not priority substances. In addition, the Atlantic RBCA states an EQS value of 1 ng/L of dieldrin for surface freshwater.

Dieldrin has also been detected in the Chinese environment, such as in soil, air, and water, as well as in wild animals [25,26,27,28,29]. In China, dieldrin was never produced on an industrial scale and was not used as a pesticide in agriculture [30]. However, previous studies have unequivocally demonstrated its presence, indicating the long-range transport of dieldrin. Recent research which was conducted in two sediment cores from Lake Fuxain, the largest deep freshwater lake in China, has found that dieldrin might pose a potential threat to exposed organisms [31]. Dieldrin is not included in China’s environmental quality standards for surface water (GB3838-2002) [32]. A maximum concentration of dieldrin, 470 ng/L, was identified using acute toxicity data for domestic aquatic organisms in China [33]. However, dieldrin in aquatic environments generally occurs at low concentrations, and it tends to be found at high levels within living organisms, which is harmful and toxic. Therefore, it is necessary to identify the long-term concentrations of dieldrin for the protection of aquatic life. Moreover, the established water quality standards in China do not consider the trophic magnification of chemical substances and their effects at higher trophic levels. Data on wildlife are urgently needed to assess the risks posed by persistent, bioaccumulative and toxic chemicals (PBTs) to birds or mammals in Chinese aquatic systems and to support national policy-making decisions.

Traditional water quality criteria (WQC) derived from water-based toxicity that do not consider bioaccumulation factors (BAFs) make it challenging to provide comprehensive protection for aquatic life. In addition, multiple routes of exposure (e.g., diet, sediment, water) may impact the bioavailability of these substances. Accordingly, adopting a tissue-based method for deriving aquatic life criteria is recommended [34]. There are some advantages to using tissue concentrations or residues as the dose metric to characterize toxicity: species differences in bioaccumulation and time to effect are less important factors and exposure over time and space is integrated [35]. This method has been used to derive criteria for mercury, selenium, DDT and TCDD [36,37,38,39].

The aim of this study is to determine the WQC for dieldrin in order to protect aquatic organisms and aquatic-dependent wildlife using tissue-based toxicity data to better manage the environment. This study gives new insight into the ecological risk of dieldrin and provides a scientific foundation for enhancing WQC determination systems in China by serving as a reference for the derivation of bio-accumulative substances.

## 2. Methods

### 2.1. Toxicity Data Screening

The sources of tissue-based toxicity of aquatic organisms for dieldrin are the Environmental Residue Effects Database, the ECO-TOX database and the published literature. For wildlife, tissue-based toxicity data were obtained from the literature compiled by the USEPA. The regions of different species were mainly defined according to the Global Biodiversity Information Facility (https://www.gbif.org/, accessed on 18 September 2024). A tissue-based approach needs to be developed based on ecologically relevant endpoints that protect populations [35]. Thus, data selection is guided by the following principles: (1) internationally recognized, indigenous and introduced species, including freshwater invertebrates, vertebrates and wildlife were selected; (2) the endpoints of the effect include morality, developmental growth, reproduction, behavior and accumulation; (3) the no-observed effect concentration (NOEC) was selected as the test endpoint to develop chronic criteria; and (4) residue data are obtained from whole-body measurements.

### 2.2. Derivation of Tissue-Based Criteria

Provided that sufficient valid toxicity data are available, the species sensitivity distribution (SSD) method was selected to obtain a baseline or toxicity reference value. This probability distribution function assumes that sensitive species can be characterized by statistical distributions. The SSD method is widely used to obtain water quality benchmarks, characterizing the impacts of chemical contaminants on water quality and/or for ecological risk assessment [40]. Designating the most sensitive species, a hazard concentration, i.e., the 5th percentile of the SSD which protects 95% of organisms, is used to set the criteria [41].

The tissue-based criteria (TBC) refers to criteria generated from toxicity data reported as concentrations in tissues of the target organisms or their diet (in wildlife) [42]. Tissue-based toxicity metrics are developed using the same methodology as that for external concentration-based toxicity tests [35]. In the case of aquatic-dependent wildlife, toxicity data of avian or mammalian species expressed as tissue concentrations per day are used to generate HC_5_, representing the tolerable daily intake (TDI). In order to obtain the WQC, tissue concentrations need to be represented as concentrations in the aquatic diet. The tissue reference value (TRV) for aquatic-dependent wildlife is calculated using the TDI in conjunction with the daily food ingestion rates (FIs) and the body weights (BWs) of wildlife species [43], as shown in Equation (1).

(1) $$TRV=\frac{TDI}{FI:BW}$$

where the TDI is derived using the SSD method using tissue-based toxicity data (mg/kg) and FI:BW is the ratio of food ingestion rate to body weight of the representative wildlife species in China, which were selected from the literature.

### 2.3. Obtaining the WQC for Dieldrin

In order to translate the tissue criterion to corresponding water concentrations, it should be divided by the relevant bioaccumulation factors (BAFs) determined for each representative species [42]. Bioaccumulation can be viewed as the combination of bioconcentration and food uptake as it occurs when chemicals accumulate in aquatic organisms through multiple exposure routes, including dietary, respiratory, and dermal absorption [44]. The BAF is the ratio of the concentration of a chemical in an organism to that in the environment. Fish have among the highest bioaccumulation rates for dieldrin and higher trophic levels in aquatic ecosystems. From there, the empirical model based on field-derived BAFs is used to assess bioaccumulation potential and to translate tissue-based criteria to water concentrations [34]. There are no available site-specific BAF measurements obtained though paired water concentrations and fish tissue data for the recommended representative species. Herein, a generic BAF model for fish development by Arnot and Gobas [45] was used to estimate the BAF of dieldrin. The model provides predictions of BAF for fish species in the lower, middle and upper trophic levels of aquatic food webs and can be used to predict dietary concentrations for higher trophic level predators (e.g., birds and mammals) via the fish in their diet. The model is a quantitative structure–activity relationship (QSAR), with input parameters consisting only of the octanol–water partition coefficient (Kow) of the chemical and, if available, the metabolic transformation rate constant. Since the metabolic transformation rate constant was not available, the log_10_Kow value of 5.4 for dieldrin, determined using the “slow-stirring” method [46], was used as the input parameter for the BAF–QSAR model. Then, using Equation (2), TBC or TRV were converted to the WQC with the estimates of BAF, expressed as the concentration in the water.

(2) $$WQC_{aqua}\left(WQC_{wild}\right)=\frac{TBC\left(TRV\right)}{BAF}$$

where WQC_aqua_ and WQC_wild_ represent the protection of aquatic life and aquatic-dependent wildlife, respectively. TBC is derived from tissue–based toxicity data using the SSD method (mg/kg), TRV is calculated using Equation (1), and BAF is the bioaccumulation factor (L/kg).

### 2.4. Ecological Risk Assessment

The available literature data on concentrations of dieldrin in China were used for environmental risk screening via the quotient method. Risk quotients (RQs) are the ratio of points estimates of exposure and toxicity. Exposure refers to actual monitored or model-estimated environment concentration. The toxicity refers to an effect level such as the predicted no-effect concentration (PNEC), which is the ecological risk threshold predicted to have no adverse effects on organisms. RQs were calculated as shown in Equation (3):

(3) RQ=C/WQC

where C is the measured environmental concentration collected from the literature; WQC for dieldrin is derived from Equation (2), which expresses the ecological risk threshold. Then, it is compared to the EPA’s levels of concern (LOCs). According to the EPA’s risk assumptions, the value of the LOC is 1 for chronic risk. A resulting RQ below the value of 1 indicates no chronic risk concern.

### 2.5. Data Analysis

The current study employs the R package “ssdtools” to generate the SSD [47] using R version 4.1.0 [48]. We utilized the “ssd_fit_dists” function using maximum likelihood to fit the distribution and the “ssd_gof” function to assess the goodness of fit. Using bootstrapping to obtain confidence intervals, the “ssd_hc” function was used to estimate the model-averaged 5% concentration; that is, the HC_5_ value. Together with the original data, the predictions were plotted using the “ssd_plot” function. The model BAF-QSAR v1.1 [45] coded in a Microsoft Excel workbook was used to obtain the BAF. The data screening and derivation of WQC were also carried out using Microsoft Excel 2019 for Windows.

## 3. Results and Discussion

### 3.1. WQC for the Protection of Aquatic Life

**Figure 1:**
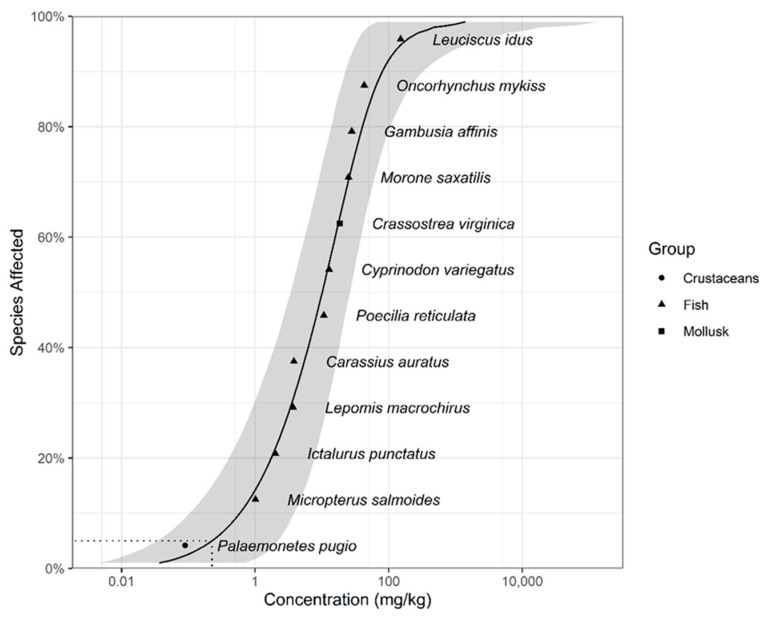
Species sensitivity distribution for dieldrin for aquatic organism tissue-based toxicity data. The model-averaged 95% confidence interval is indicated by the shaded band and the model-averaged 5% hazard concentration (HC5) by the dotted line.

Table 1 presents toxicity data obtained from database for the dieldrin levels present in aquatic life. The selected species, including ten fishes, one crustacean and one mollusk, met the minimum data requirements. As shown in Figure 1, the SSD curve with the confidence interval was simulated from collated data. The TBC is 0.227 mg/kg (wet weight; WW), derived from estimated model-averaged predictions of 5% hazard concentrations with parametric bootstrapping.

Since data on dieldrin in the environment and biota are insufficient, the model approach was used to obtain the BAF. The estimation of the logarithm of BAFs to base 10 in the lower, middle and upper trophic levels of fish species are 4.77, 5.09 and, 5.66, respectively. The BAF predictions are considered generic, and they are not considered to be for a particular species of fish. Moreover, Arnot and Gobas [49] reviewed the status of bioaccumulation evaluations for organic chemicals in aquatic systems and summarized regression statistics for different organism classes before and after the confidence assessment on the reviewed data. In agreement with their empirical model, the log_10_BAF value of 4.77 was used to derive the WQC for dieldrin. The calculated BAF for fish is 58,884.37 L/kg. Using Equation (3), the calculated WQC for aquatic organisms is 3.86 ng/L.

**Table 1:** Tissue-based toxicity data of aquatic life for dieldrin.

Species	Common Name	NOEC (mg/kg, WW *)	Taxa	Reference
*Palaemonetes pugio*	Grass shrimp	0.09	Crustaceans	[50]
*Micropterus salmoides*	Largemouth bass	1.01	Fishes	[51]
*Ictalurus punctatus*	Channel catfish	2	Fishes	[52]
*Lepomis macrochirus*	Bluegill	3.7	Fishes	[53]
*Carassius auratus*	Goldfish	3.8	Fishes	[53]
*Poecilia reticulata*	Guppy	10.7	Fishes	[54]
*Cyprinodon variegatus*	Sheepshead minnow	12.8	Fishes	[50]
*Crassostrea virginica*	Eastern oyster	18.6	Mollusks	[55]
*Morone saxatilis*	Striped bass	25	Fishes	[56]
*Gambusia affinis*	Mosquito fish	28	Fishes	[57]
*Oncorhynchus mykiss*	Rainbow trout	43	Fishes	[58]
*Leuciscus idus*	Golden ide	151	Fishes	[59]

* WW = wet weight.

### 3.2. WQC for Protection of Aquatic-Dependent Wildlife

**Figure 2:**
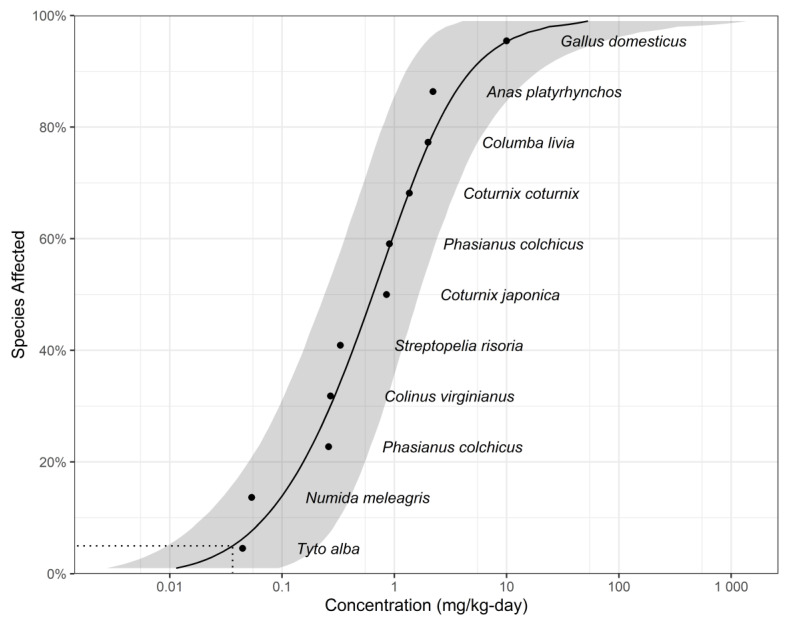
Species sensitivity distribution for dieldrin for avian tissue-based toxicity data. The model-averaged 95% confidence interval is indicated by the shaded band and the model-averaged 5% hazard concentration (HC5) by the dotted line.

**Figure 3:**
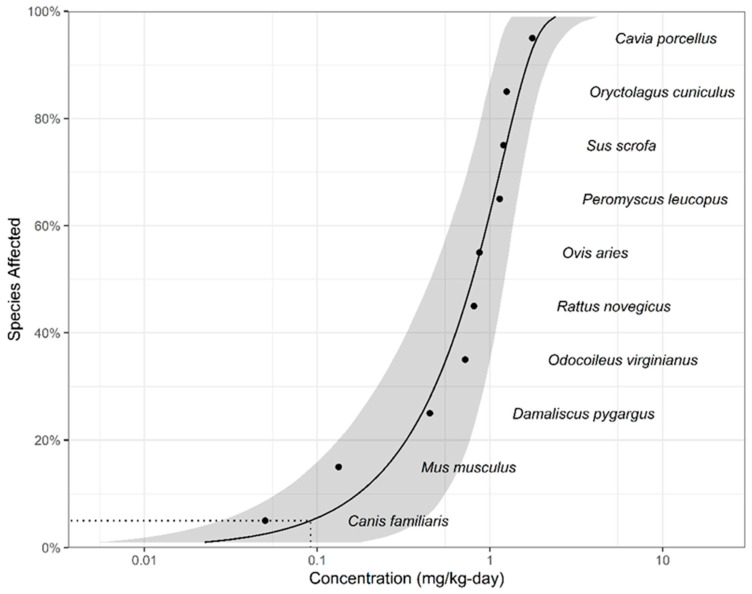
Species sensitivity distribution for dieldrin for mammalian tissue-based toxicity data. The model-averaged 95% confidence interval is indicated by the shaded band and the model-averaged 5% hazard concentration (HC5) by the dotted line.

Table 2 presents the toxicity data for dieldrin of wildlife, including 11 avians and 10 mammalians which met the minimum data requirement. As shown in Figure 2 and Figure 3, the SSD curves with confidence intervals were simulated for each taxa group from the collated data, and 5% hazard concentrations were calculated. The TDIs for avians and mammalians are 0.0363 and 0.0914 mg/kg per day, respectively. The highest FI:BW value among representative avian species in China is 0.43 [60], and the higher ratio of FI to BW among representative mammalian species in China is 0.5 [61,62,63]. The TRVs are 0.08442 and 0.1828 mg/kg WW, respectively. Using Equation (3) and the previous calculated BAF for fish, the calculated WQC are 1.4 and 3.10 ng/L, respectively. To better protect aquatic-dependent wildlife, the smallest WQC (1.40 ng/L) was selected as the dieldrin WQC for protecting aquatic-dependent wildlife in China.

Considering the bioaccumulation of dieldrin in piscivorous food webs, the threshold contaminant body burden in wildlife was calculated, which was then back-calculated to an equivalent concentration in fish or water using the combination of food and chemical assimilation efficiencies and bioconcentration/bioaccumulation factors. For wildlife criteria derived from dietary toxicity data, BAFs would be applied and appropriately weighted for each component of the aquatic diet of the representative wildlife species [42]. Using this method, the dieldrin wildlife value for waters within the Great Lakes Basin was determined to be 0.071 ng/L [23]. According to protocol used in the Canadian Tissue Residue Guidelines for the Protection of Wildlife that Consume Aquatic Biota [43], reference concentrations are calculated using information on BW and FI for wildlife species as well as the TDI derived from toxicity studies; thus, the result can be compared to the generic tissue residue guideline developed to protect all wildlife. For substances with a high potential for bio-magnification within food chains, it is important that the guideline used for lowest reference value is applied to the highest aquatic trophic level (e.g., level 4 fish) in order to protect predators feeding at that level.

Since species-specific and site-specific data for dieldrin were not available in China, we selected the highest ratio of FI to BW of representative wildlife species to calculate the TRV from TDI and used BAF for fish to convert the TRV to water concentration. Dieldrin was found in a fish liver sample (0.07 μg/g WW) collected from aquaculture cages in coastal waters of Xiamen [84] and in various organs and tissues of Indo-Pacific humpback dolphins from the Pearl River Estuary, with values ranging from 0.74 to 6.8 ng/g WW [28]. Our WQC is much higher than that of the Great Lakes Basin, but compared to residue records in biota, it should be effective for protecting wildlife.

### 3.3. Risk Assessment

Studies have shown that chronic exposure to dieldrin can cause harm to aquatic organisms and wildlife [10,85]. Therefore, it is important to assess the ecological risk of dieldrin in aquatic environments. The data on the presence of dieldrin in surface water from the last dozen years, identified by the literature review and the ecological risk assessment, are shown in Table 3. Samples were collected from the Yangtze River, the Qinhuai River and the Xuanwu Lake in Nanjing [27] and the Shaying River Basin [86], and the mean concentrations of dieldrin were 1.31, 2.32, 4.38 and 4.6 ng/L, respectively. RQ values were calculated separately using WQC for the protection of aquatic life and wildlife. Results ranging between 0.34 and 3.29 were observed, suggesting that dieldrin poses an ecological risk to both aquatic organism and wildlife in the Xuanwu Lake and the Shaying River Basin, poses a potential ecological risk to avians in the Qinhuai River and poses no chronic risks in the Yangtze River.

Regarding the sampling approach, as the timing and site selection were unable to adequately capture the periodic occurrence of pesticides or investigate surface waters particularly susceptible to pesticide risks, this may contribute to an inappropriate estimation of risk [87]. Since dieldrin has been banned and is not used in China, its main source is environment migration. With hydrophobicity and low water solubility, dieldrin is prone to binding to organic materials [1]. Research on the distribution of organochlorine pesticide pollution in Indonesia revealed that sediments showed higher organochlorine concentrations than water, mollusks or fish [15]. Sediment is still a potential source and is rarely detected. It has been demonstrated that the concentration in sediments can be accurately predicted by multiplying the concentration in water by the chemical’s organic carbon partition coefficient [22]. Thus, detecting dieldrin concentrations in water is important for better understanding its ecological risk to integral aquatic ecosystems.

## 4. Conclusions

This study set out to determine the WQC for dieldrin using tissue-based toxicity data. In this study, the estimated BAF of 58,884.37 L/kg was used to obtain the WQC. The tissue-based criteria obtained using screened dieldrin toxicity data and the SSD method are 0.227 mg/kg (WW) for aquatic life and 0.08442 and 0.1828 mg/kg of food per day (WW) for avian and mammalian species, respectively. Using the statistics presented above, the dieldrin WQC needed for the protection of aquatic organisms and wildlife in China are 3.86 and 1.4 ng/L, respectively. This study is limited by the lack of information on toxicity data and BAF measurements of dieldrin. Notwithstanding these limitations, this work contributes to furthering the protection of aquatic organisms and wildlife from the bioaccumulation of dieldrin, supporting environmental management and risk assessment in China. The results also enhance China’s water environment criterion system, enabling the development of an “ecological water civilization”. This research has also shown that dieldrin levels in surface water in parts of China may pose potential ecological risks to aquatic organisms and wildlife, especially avian species. These findings are cause for concern of the presence of dieldrin residue in Chinese aqueous environments. Meanwhile, considerably more work needs to be carried out to monitor dieldrin levels in surface water environments in China.

## Figures and Tables

**Table 2:** Tissue-based toxicity data of aquatic-dependent wildlife for dieldrin.

Species	Common Name	NOEC (mg/kg bw/day, WW) *	Taxa	Reference
*Tyto alba*	Barn owl	0.0445	Avian	[64]
*Numida meleagris*	Crowned guinea fowl	0.0537	Avian	[65]
*Phasianus colchicus*	Ring-necked pheasant	0.26	Avian	[66]
*Colinus virginianus*	Bobwhite quail	0.27	Avian	[67]
*Streptopelia risoria*	Ring dove	0.331	Avian	[68]
*Coturnix japonica*	Japanese quail	0.852	Avian	[69]
*Phasianus colchicus*	Pheasant	0.905	Avian	[70]
*Coturnix coturnix*	Quail	1.36	Avian	[71]
*Columba livia*	Homing pigeon	2	Avian	[72]
*Anas platyrhynchos*	Mallard	2.21	Avian	[73]
*Gallus domesticus*	Chicken	10	Avian	[74]
*Canis familiaris*	Dog	0.05	Mammalian	[75]
*Mus musculus*	Mouse	0.133	Mammalian	[76]
*Damaliscus pygargus*	Blesbuk	0.449	Mammalian	[77]
*Odocoileus virginianus*	White-tailed deer	0.72	Mammalian	[78]
*Rattus novegicus*	Rat	0.81	Mammalian	[75]
*Ovis aries*	Sheep	0.87	Mammalian	[79]
*Peromyscus leucopus*	White-footed mouse	1.14	Mammalian	[80]
*Sus scrofa*	Pig	1.20	Mammalian	[81]
*Oryctolagus cuniculus*	Rabbit	1.25	Mammalian	[82]
*Cavia porcellus*	Guinea pig	1.76	Mammalian	[83]

* bw = body weight; WW = wet weight.

**Table 3:** RQ estimations.

Location	Conc/(ng/L)	RQ_Aqua	RQ_Wild	Sampling Time	Reference
Yangtze River, Nanjing	1.31	0.34	0.94	May to July 2016	[27]
Qinhuai River, Nanjing	2.32	0.60	1.66		
Xuanwu Lake, Nanjing	4.38	1.13	3.13		
Shaying River Basin	4.60	1.19	3.29	November 2013	[86]

## Data Availability

The original contributions presented in this study are included in the article. Further inquiries can be directed to the corresponding author.
